# A Novel Miniature and Selective CMOS Gas Sensor for Gas Mixture Analysis—Part 2: Emphasis on Physical Aspects

**DOI:** 10.3390/mi11060587

**Published:** 2020-06-11

**Authors:** Moshe Avraham, Sara Stolyarova, Tanya Blank, Sharon Bar-Lev, Gady Golan, Yael Nemirovsky

**Affiliations:** 1 Electrical Engineering Department, Technion—Israel Institute of Technology, Haifa 32000, Israel; smoa@technion.ac.il (M.A.); ssstolya@technion.ac.il (S.S.); tblank@technion.ac.il (T.B.); sharonb@technion.ac.il (S.B.-L.); 2 Department of Electrical Engineering and Electronics, Ariel University, Ariel 40700, Israel; gadygolan@gmail.com

**Keywords:** CMOS-SOI-MEMS gas sensor, catalytic micro hot-plate, thermal gas sensor, MEMS simulations and modeling

## Abstract

This is a second part of the paper presenting a miniature, combustion-type gas sensor (dubbed GMOS) based on a novel thermal sensor (dubbed TMOS). The TMOS is a micromachined CMOS-SOI transistor, which acts as the sensing element and is integrated with a catalytic reaction plate, where ignition of the gas takes place. Part 1 focused on the chemical and technological aspects of the sensor. In part 2, the emphasis is on the physical aspects of the reaction micro-hot plate on which the catalytic layer is deposited. The three main challenges in designing the hot plate are addressed: (i) How to design a hot plate operating in air, with a low thermal conductivity; (ii) how to measure the temperature of the hot plate during operation; (iii) how to reduce the total consumed power during operation. Reported simulated as well as analytical models and measured results are in good agreement.

## 1. Introduction

There is an ongoing effort to fabricate miniature, low cost, sensitive, and selective gas sensors for domestic and industrial uses [1,2,3,4,5,6,7,8,9,10,11]. The miniaturization and reduced power consumption of gas sensors allow for a wide range of application in wearable and portable devices, such as mobile and smart phones. Recently we reported a miniature, combustion type gas sensor (dubbed GMOS) based on a thermal sensor, where a micro-machined CMOS-SOI transistor acts as a sensing element and is integrated with catalytic reaction plate and embedded heater [12,13,14,15,16]. The suspended transistor-dubbed TMOS exhibits extremely high sensitivity to the change of the temperature [17,18,19,20,21,22]. Ref. [16] presents Part 1 of the present paper, which emphasizes the GMOS chemical performance modeling, as well as the two deposition techniques of Pt catalytic layer suitable for wafer level processing, magnetron sputtering, and nanoparticle inkjet printing.

The present paper, Part 2, focuses on the physical modeling of the hot catalytic plate of the GMOS sensor. The three main challenges in designing the hot plate are addressed: (i) How to design a hot plate operating in air, with a low thermal conductivity (Section 2); (ii) how to measure the temperature of the hot plate during operation (Section 3); (iii) how to reduce the total consumed power during the sensing (Section 4).

In order to understand the nature of the above challenges let us bear in mind the following:

(i) The catalytic layer requires heating to the operation ignition temperature as well as periodic thermal refreshment at ~400 °C in order to avoid its degradation. At the same time, a large area reaction plate is preferred since the measured signal scales with the area and a larger area facilitates the deposition of the catalytic layer. It is shown in Section 2 that there is a trade-off between these two requirements.

(ii) The selectivity of the GMOS is achieved by monitoring the ignition temperature of the sensed gas, denoted by T* and discussed in Part 1. The temperature control as well as its monitoring is therefore of paramount importance.

(iii) IoT applications and mobile applications require battery operation. Hence, reducing the overall power during operation is essential.

**Figure 1:**
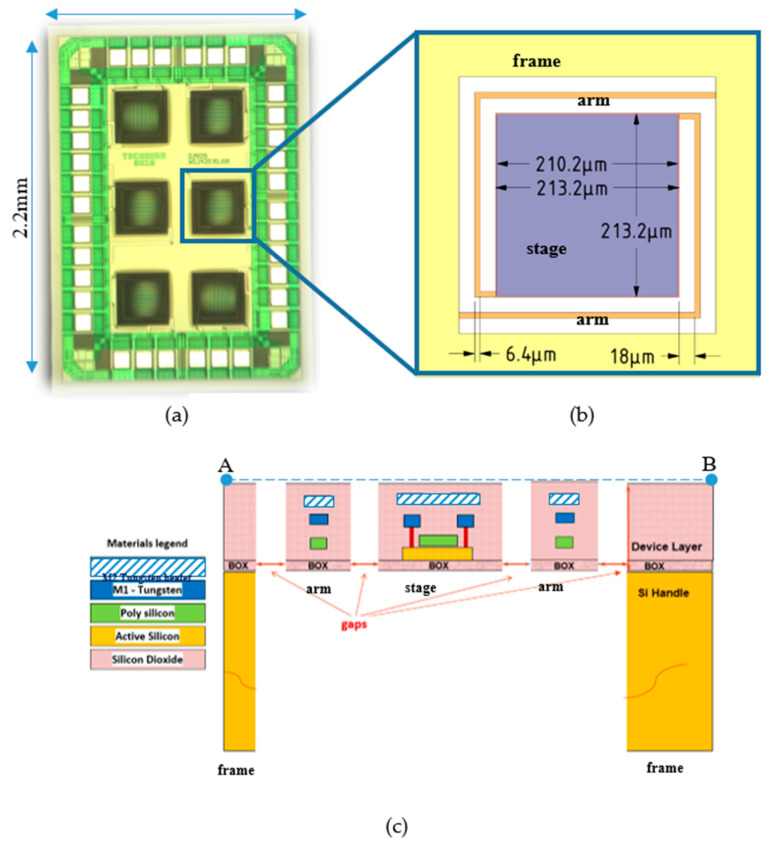
(**a**) Microscopic overview of the GMOS sensor die; (**b**) schematic overview of a GMOS pixel with typical dimensions. (**c**) Schematic cross section of the GMOS single pixel (DUT-device under study).

The main simulation tool of the present study is ANSYS FLUENT [23]. The simulations are corroborated by analytical modeling, which also gives a better physical insight. The results are confirmed by measurements. The device under study (DUT) is shown in Figure 1 and more details can be found in Part 1 [16]. It should be noted that the design is a compromise between a small die, with overall dimensions of less than 4 mm^2^ and a pixel area that facilitates the deposition and increases the signal, thus enabling high sensitivity even at low concentrations of the detected gases.

## 2. Hot Plate Operating in Air, with a Low Thermal Conductivity

### 2.1. Modeling Supported by Simulations

**Figure 2:**
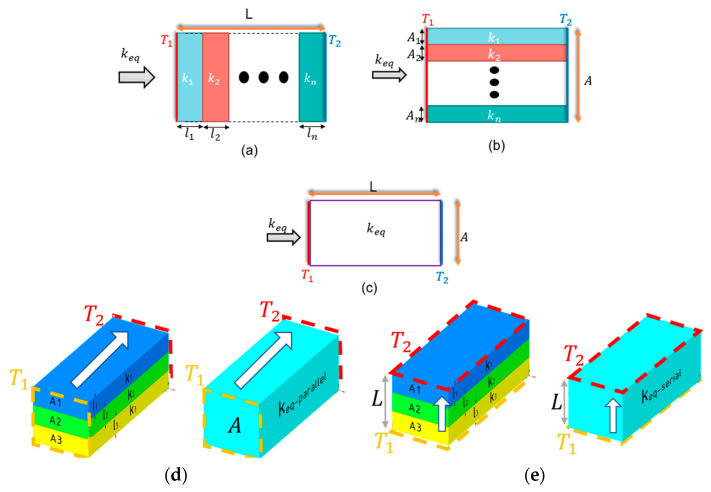
(**a**) Paralleled n materials. (**b**) Serialed n materials. (**c**) The equivalent solid. (**d**) 3D model demonstration of the parallel equivalent thermal conductivity, assuming $T_{1}>T_{2}$. (**e**) 3D model demonstration of the serial equivalent thermal conductivity, assuming $T_{1}>T_{2}$.

As shown in Figure 1, the device includes several layers. Small features and layer thickness require denser mesh and more elements, which obligate large computations resources and running simulation time. Two modeling methods were used to reduce the number of elements: (i) layers with small thickness were neglected by the assumption that the materials have similar thermal properties. (ii) Calculating the solid equivalent thermal conductivity, $k_{eq}$, which is direction dependent $\left(k_{eq_{x}},k_{eq_{y}},k_{eq_{z}}\right)$, with Equation (1a,b) of parallel and serial materials and substituting the solids with one equivalent solid in the 3D model as can be shown in Figure 2.

(1) $$\left(a\right)\;k_{eq_{serial}}=L\cdot {\left(\overset{n}{\underset{i=1}{\sum }}\left(\frac{l_{i}}{k_{i}}\right)\right)}^{-1}\;\left(b\right)\;k_{eq_{parallel}}=\frac{1}{A}\cdot \overset{n}{\underset{i=1}{\sum }}k_{i}A_{i}$$

where *n* is the number of solids connected. *A* and $A_{i}$ are the areas of the solid that the heat flux go through. *L* and $l_{i}$ are the distances the heat flux go through (note: $A=\sum_{i=1}^{n}A_{i}$ and $L=\sum_{i=1}^{n}l_{i}$) and $k,\frac{\mu W}{K}$ is the thermal conductivity.

**Figure 3:**
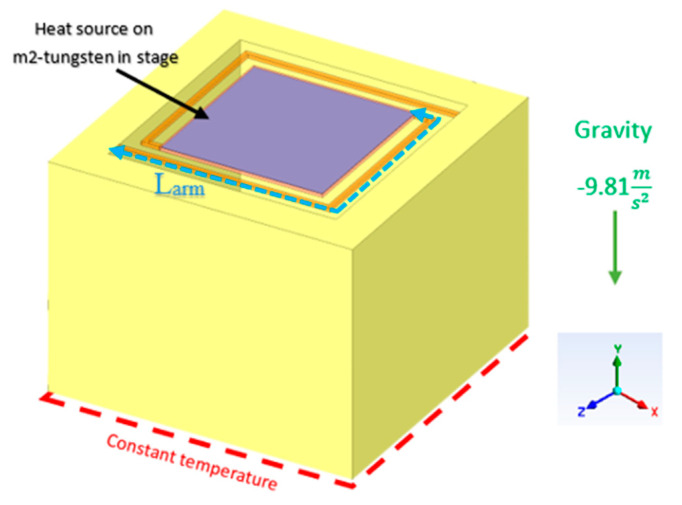
The 3D ANSYS model.

As can be seen in Figure 3, the sensor is thermally insulated from the frame by holding arms. The thermal conductance, $G_{th}$, is determined by the thermal conductance of the sensor solid parts and by the air conduction around the plate. A 3D Finite Elements Analysis software is required for modeling and simulation the thermal conductance of the sensor.

Boundary and operating conditions to the 3D model were set. The bottom of the device was set to a constant temperature. A power heat source was set to the tungsten heater in the stage. The actual device had a meandered heater, however in the simulation we used a single metal plate. We assume that the volume of the film produces a power $P_{Joule}=\frac{V_{Heater}^{2}}{R_{Heater}\left(T\right)}$, in that way we enforce the film to produce the same heating power as a meander shape heater with resistance of $R_{Heater}\left(T\right)$ and applying voltage $V_{Heater}$. Since the meander shape is uniform across the plate, the results are very similar. The air density is determined by the ideal gas law. The air flow is caused by natural convection. The air flow direction is in the y direction on the ANSYS Fluent software. The solid material thermal properties were provided by the FAB (Table 1).

The thermal conductance of a solid can be calculated by:

(2) $$G_{th}=\frac{kA}{L}$$

where *A* is the area of the solid that the heat flow, which caused by a temperature difference, go through and *L* is the solid length. For example, in our DUT (device under study) a temperature difference develops on the arm and L is the length of the arm from the frame to the plate as shown in Figure 3. The thermal conductivity of the arm can be calculated using Equation (1).

**Figure 4:**
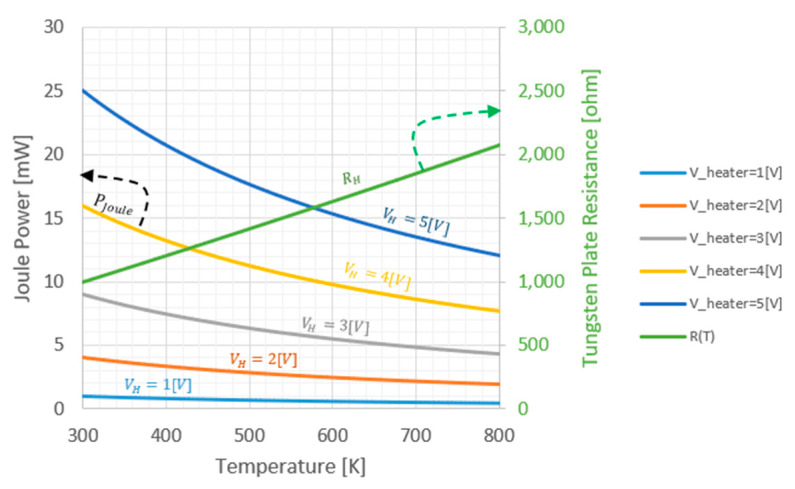
Joule power on tungsten heating resistor vs. temperature for *R*_0_ = 1000 ohm.

**Figure 5:**
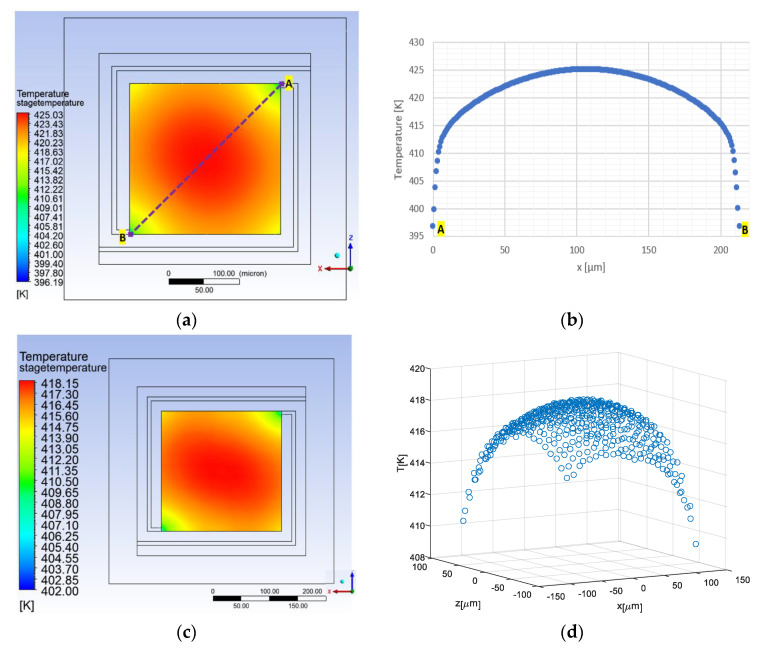
Steady-state thermal simulation results of the DUT design for $V_{heater}=3\;V$, and $R_{heater}=1000\;\mathrm{ohm}$: (**a**) Distribution of the temperature over the bare plate. (**b**) The temperature of the plate among the line between point x and y in Figure 5a. (**c**) 2-D temperature distribution over plate with platinum catalytic layer and silicon nitride. (**d**) 3-D temperature distribution over the plate with platinum and silicon nitride layers.

A Joule heating was applied to the heater resistor on top of the plate $P_{joule}=\frac{V_{applied}^{2}}{R_{heater}\left(T\right)}$. The tungsten heater resistance dependency is the following:

(3) $$R_{heater}\left(T\right)=R_{0}(1+TCR_{1}\left(T-T_{0}\right)+TCR_{2}{\left(T-T_{0}\right)}^{2}$$

where $R_{0}$ is the initial resistance at the room temperature, and $TCR_{1}=2.05\times 10^{-3}\;K^{-1}$ and $TCR_{2}=0.2\times 10^{-6}\;K^{-2}$ are temperature coefficients of resistance parameters, which define the change of the resistance due to the change of the temperature. The *TCR* values were provided by FAB (XFAB, Erfurt). In this work, three values of resistors were studied. Figure 4 shows the resistance and the Joule heating power dependencies for R=1000 ohm. Figure 5 exhibits the uniformity of the temperature across the plate. The simulation was done for a bare stage as well as with silicon nitride layer of 0.75 µm and platinum catalytic layer of 0.5 µm on top of the stage. Furthermore, this simulation took into consideration the temperature dependency of the thermal conductivity of the air which increases approximately linearly with the temperature. As can be seen, the temperature variation on the stage occurs mostly at the corners, especially at the holding arms contacts. The presence of silicon nitride and platinum catalytic layers on top of the stage significantly improves the temperature uniformity. In addition, to compensate the temperature variations a non-uniform resistor should be designed with local variations of the resistance at the corners. At the corners, a narrower meander which provides locally a higher resistor value and hence higher local Joule heating, may further increase the temperature uniformity.

Note: The Joule power, which develops is $P_{Joule}=\frac{V_{Heater}^{2}}{R\left(T\right)}$. Since the *TCR* (temperature coefficient of resistance) is positive for tungsten resistor, applying voltage rather than current is mandatory to avoid thermal run away. In contrast, since, $P_{Joule}=I^{2}R\left(T\right)$, if the current is applied, a positive feedback develops, and the Joule power may increase too much thus causing burning of the heater.

The temperature increase of the hot plate due to the Joule heating is given by:

(4) $$\Delta T_{J}=\frac{P_{Joule-heating}}{G_{th}}$$

**Figure 6:**
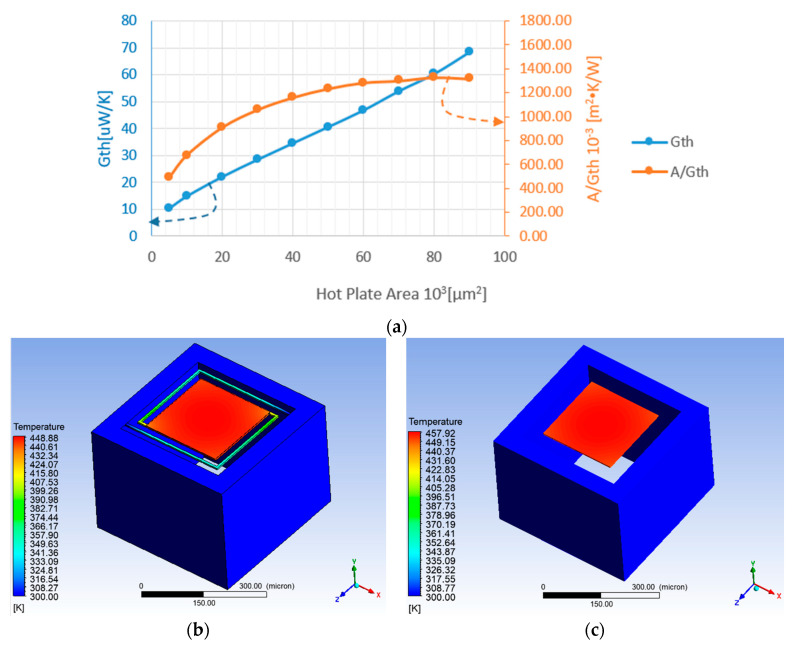
(**a**) Ideal hot plate thermal conductance and the FOM Area/*G_th_*. Vs. stage area for $V_{heater}=3\;V$, and $R_{heater}=1000\;\mathrm{ohm}$. The hot plate is ideal since the effect of the holding arms is neglected. (**b**) Steady state simulation temperature result of the DUT with holding arms, with the overall following dimensions: Plate area: $45.45\times 10^{3}$ µm^2^; Holding arms width 6.4 µm: Gap 18 µm; for applied heater voltage of 3 V. (**c**) Steady state simulation temperature result of the DUT without holding arms.

For refreshment of the catalytic plate, a temperature difference of ~350–400 °C is required [16,24]. Accordingly, the required Joule heating power is $P_{Joule-heating}=400\cdot G_{th}$ and for *G_th_* of 50 µWatt/K it is *P_Joule-heating_* = 400 × 50 ×10^−6^ = 20 mWatt. For battery operation, this sets a practical limit to the plate area, as shown below (see Figure 6).

### 2.2. Simulations Supported by Modeling

In this section, the thermal simulations have been performed to determine thermal conductance, and optimal plate area for a fixed pixel size. In the simulations, the heating power was applied to the reaction plate, and the temperature increase Δ*T* of the plate was obtained. Then, to determine the *G_th_*, the applied power was divided by the average Δ*T* (according to the Formula (4)). In Part 1 of the present paper [16], it was shown that the sensor response $v_{sig}\left(V\right)$ is proportional to the reaction plate area and inversely proportional to the *G_th_*.

(5) $$v_{sig}=\left(\frac{dV_{DS}}{dT}\right)\cdot C_{g}\cdot \left(\frac{1}{k_{s}}+\frac{\delta }{D}\right)\cdot A_{plate}\cdot \left(\frac{\Delta H_{C}}{G_{th}\cdot N_{A}}\right)$$

where *V_DS_*—drain-source voltage of the transistor, V; *T*—transistor temperature, K; $k_{s}$—reaction rate, m/s; *C_g_*—gas concentration in air, molecules/m^3^; *D*—gas diffusion constant, m^2^/s; *δ*—stagnant film thickness, m; ∆*H_C_*—combustion enthalpy, Joule/mole; *N_A_*—Avogadro number, 1/mole.

In order to achieve a higher signal from the chemical reaction a larger hot-plate area is required [16], but on the other hand the enlargement of the hot-plate area increases the thermal conductance, as shown in Figure 6. It shows that as A—the plate area increases, *G_th_* also increases almost linearly, and thus the required Joule power needed for heating the plate for operation and refreshment also increases. Therefore, we have defined *A_hot-plate_*/*G_th_* as the figure of merit (FOM) of the hotplate since it expresses the tradeoff between the reaction efficiency and the needed power for operation and refreshment. Figure 6a shows that *A*/*G_th_* starts to saturate at the plate area of about 45 × 10^3^ µm^2^. This value was taken for the actual design of the device. However, if we print catalytic nano particles on the hot plate, then the actual surface area is larger than the physical area of the hot plate, and the latter can be further scaled down.

An ideal model with no arms with fixed pixel area of 384 × 384 µm^2^, while the reaction area of the plate varied, was simulated and the results can be seen in Figure 6. As the plate area increases the gap between the frame and the plate decreases. The FOM saturates for a hot plate of ~$250\times 250{\;\mu m}^{2}$, and a total gap (no arms) to the pixel’s frame is 67 µm.

As can be seen in Figure 6b the average temperature difference is 146.52 K and for the same model but without the solids arms, the average temperature difference is 155.9 K. The thermal conductance of the device with holding arms is $G_{th}=47.36\frac{\mu W}{K}$, and for the ideal model, without solids arms is $G_{th}=43.8\;\frac{\mu W}{K}$. By comparing the *G_th_* of Figure 6b with that of Figure 6c it can be seen that for the large hot plate the effect of the thermal conductivity of the arms is quite small.

The simulated *G_th_* may be supported by modeling, assuming that the main heat convection is upwards since hot air has lower density. Moreover, we may assume a stagnant film of air, through which the sensed gas diffuses. The temperature on the upper part of the stagnant air is the ambient temperature whereas the temperature on the lower part is determined by the hot plate. The stagnant film model is briefly considered in [25]. By assuming a stagnant film of several tens of microns denoted by *L*, the modeled *G_th_* corresponds to the simulated one since: $G_{th}=0.026\cdot \frac{A_{plate}}{L}$ *W/K*, where the air thermal conductivity is assumed. The stagnant film thickness assumed here is determined from experiments where T* is determined [16].

**Figure 7:**
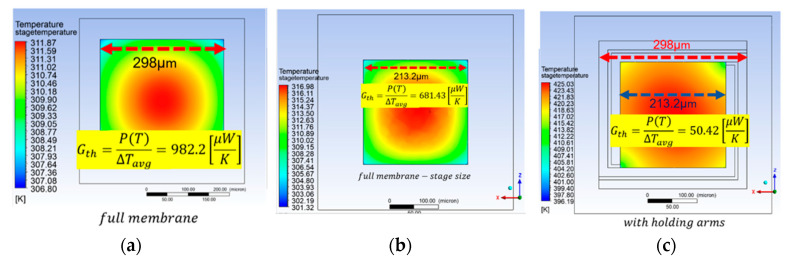
(**a**) Steady state simulation temperature results and *G_th_* for: (**a**) full membrane having a pixel size area ($298\times 298{\;\mu m}^{2})$. (**b**) Full membrane having a stage size area ($213\times 213{\;\mu m}^{2})$. (**c**) Suspended plate of DUT (pixel size $298\times 298{\;\mu m}^{2};$ stage size $213\times 213{\;\mu m}^{2}$, holding arms width 6.4 µm, gap 18 µm). $V_{heater}=3\;V$, and $R_{heater}=1000\;\mathrm{ohm}$.

In addition, we have compared our sensor design with traditional full membrane sensor-without a gap between the stage and the frame (Figure 7). The simulations show that the thermal conductance of the traditional full membrane design is more than an order of magnitude higher than that of DUT.

Assuming that in the full membrane case, the heat transfer via contact with the bulk frame dominates over that through the air, the *G_th_* (for the membrane size $213\times 213{\;\mu m}^{2}$ and thickness of 4.6 μm) can be analytically calculated as: $G_{th}\approx 4\cdot 1.4\cdot \frac{4.6\cdot 10^{-6}\cdot 213.2\cdot 10^{-6}}{10^{-6}}=550\frac{\mu W}{K}$. By adding the thermal conductance component caused by air heat transfer $G_{th}=50\;\frac{\mu W}{K}$, the total $G_{th}$ becomes about 600 $\frac{\mu W}{K}$ which is in good correspondance with the simulation results.

These results show the advantage of suspended hot plate over the traditional one in reduction of power consumption due to the decrease of thermal conductance. The specific holding arm structure of DUT provided an optimal fill factor for the given pixel size. Furthermore, the arm meandering helped for the relaxation of stresses. Although it is more difficult to fabricate, the wafer level fabrication of the devices showed almost 100% mechanical yield.

## 3. Measuring the Hot Plate Temperature during Operation

### 3.1. Background

There are several options to determine the dependence of the plate temperature as a function of the voltage applied to the embedded heating resistor; for example, by measuring on-line the resistance of the heater [26,27,28,29]. In this study, the integrated CMOS transistor, dubbed TMOS, was applied for monitoring the plate temperature as a function of the voltage applied to the heating resistors, as described below. The off-line *I*(*T*)-*V* characteristics of the TMOS were measured using a semiconductor parameter analyzer (SPA), while heating the plate by applying voltage to the heating resistor. The hot plate temperature was evaluated from the slope and the swing of the TMOS characteristics at subthreshold.

**Figure 8:**
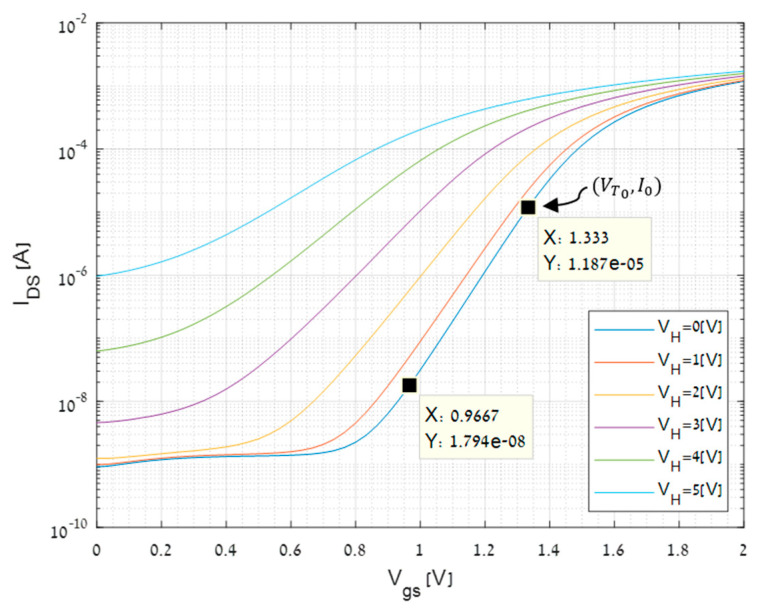
Current-voltage characteristics of the integrated TMOS (see Figure 1c) as a function of the voltage applied to the heating resistor. The current is plotted on a logarithmic scale. The relatively flat region before the exponential increase of the current is the leakage current, which increases with temperature.

Figure 8 exhibits a typical set of characteristics at subthreshold, as a function of the voltage applied to the heating resistor. The well-established exponential behavior of the current upon gate voltage at subthreshold is observed. The *I*_0_ and *V_T_*_0_ are extracted from the experimental log *I_DS_*-*Vgs* curve of the TMOS sensor operating at subthreshold. *I*_0_ and *V_T_*_0_ are obtained directly from the measured characteristic exhibited at Figure 8 when no voltage is applied to the heating resistor. The threshold voltage at subthreshold region is defined by the uppermost point where the log *I* is linear. *V_T_*_0_ = *Vgs* = 1.33 V in this case. *I*_0_ is the lowest current which is not a leakage current while maintaining the subthreshold region operation also at higher temperatures. Accordingly, we apply *Vgs* = 0.97 V in this case. The theory may be found in many textbooks, see for example the book by Sze [30]. Figure 8 also exhibits the increased leakage current as the temperature increases as well as the decreasing slope at subthreshold.

By measuring the slope and evaluating the swing (the inverse of the slope), the temperature of the hot plate is evaluated.

### 3.2. Analytical Modeling of the Measurements

The TMOS is operated at subthreshold. The well-known expression for the transistor current is used by taking into consideration the dependence of the mobility upon temperature, which follows a simple expression for *n*-mos transistors.

*T*_0_ is the reference temperature where no voltage is applied to the heater and is determined by the lab ambient temperature. In this study the temperature is assumed to be 300 K.

The transistor in the hot-plate is operating at subthreshold condition. The subthreshold current for $V_{DS}>\frac{3kT}{q}$ is:

(6) $$I_{DS}=\mu \left(T\right)C_{OX}\left(\frac{W}{L}\right){\left(\frac{kT}{q}\right)}^{2}\left(n-1\right)e^{\frac{q}{nkT}\left(V_{GS}-V_{T}\right)}$$

(7) $$\mu \left(T\right)=\mu_{0}{\left(\frac{T}{T_{0}}\right)}^{-2}$$

Combining Equations (6) and (7):

(8) $$I_{DS}\left(T\right)=I_{0}e^{\frac{q}{nkT}\left(V_{GS}-V_{T}\right)}\;\mathrm{where}\;I_{0}=\mu_{0}C_{OX}\left(\frac{W}{L}\right){\left(\frac{kT}{q}\right)}^{2}\left(n-1\right).$$

*n* is determined by the relation in Equation (9) and typically has a value of 1.4–2:

(9) $$n=1+\frac{C_{s}+C_{ss}}{C_{OX}}$$

where $C_{ss}$ is the capacitance of the fast surface states, $C_{s}$ is the semiconductor capacitance and $C_{OX}$ is the oxide capacitance.

The threshold voltage dependency on the temperature:

(10) $$V_{T}\left(T\right)=V_{T}\left(T_{0}\right)+\frac{dV_{T}}{dT}\left(T-T_{0}\right)$$

For *n*-mos $\frac{dV_{T}}{dT}$ is a negative constant and is several $\frac{mV}{K}$.

*W*/*L* is based on our design (Table 2). However, $I_{0}$ is obtained directly from the measured characteristic.

The swing *S* of the I-V at subthreshold is the inverse of the slope of the logarithmic current vs $V_{GS}$ and is given by [30]:

(11) $$S=\frac{dV_{gs}}{d\left(\log_{10}I_{ds}\right)}\approx 2.3\cdot \frac{kT}{q}\cdot n$$

The value of n is determined by the swing of the measured plot where no heating is applied, namely no voltage is applied to the heating resistor. We assume that this plot describes the behavior at the lab temperature—our reference temperature $T_{0}$. Furthermore, we assume that the dependence of *n* upon temperature is negligible. Hence, the temperature can be extracted from the swing, for various applied heater voltages. To illustrate this approach, please refer to Figure 8, assuming that the device is operated at *Vgs* = 1 V. The evaluated temperatures for the applied heater voltages are shown in the Table 3.

### 3.3. Simulations

**Figure 9:**
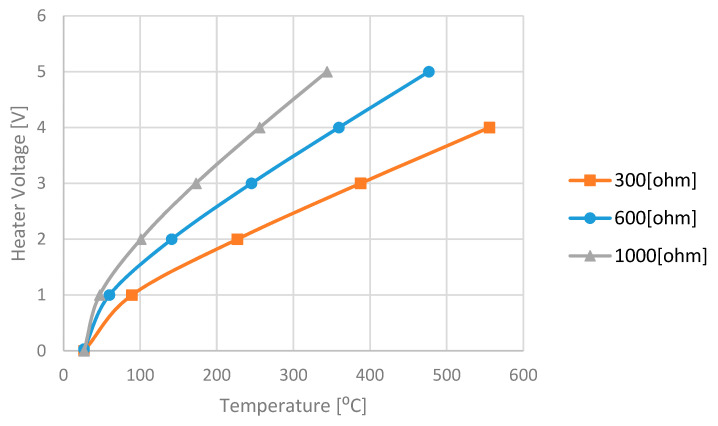
The hot-plate simulation results: applied heater voltage vs. the steady-state average hot plate temperature for different *R_heater_* values.

In this study, the 3D hot plate temperature including the holding arms, was modeled and simulated in ANSYS fluent software [23] as a function of the heater voltage for several values of heating resistors (Figure 9).

**Figure 10:**
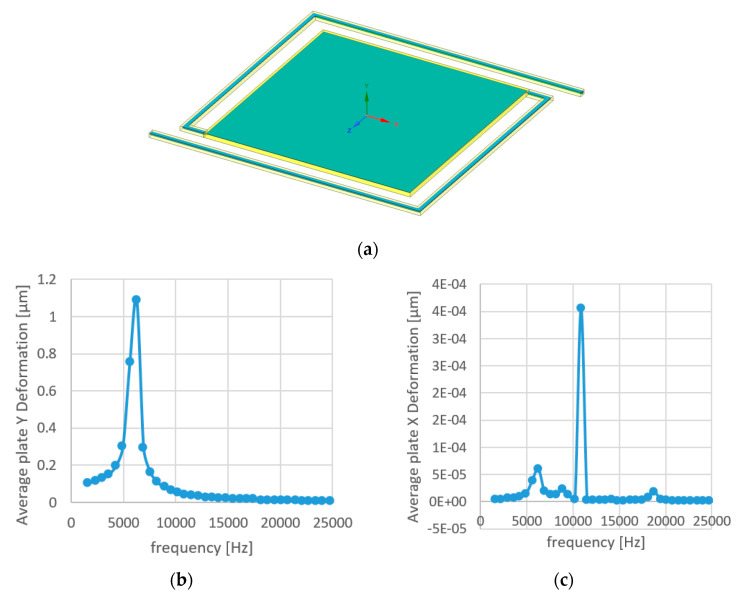
(**a**) The mechanical model, assuming vacuum and fixed support on the arm edges. (**b**,**c**) Average deformation of the plate versus frequency for applied force of F = (0,−0.1 µN,0) on the stage in Y (**b**) and X (**b**) directions.

Mechanical simulations with modal analysis and harmonic response have been made on the DUT design of Figure 1c. In order to evaluate the resonance frequencies, we assumed vacuum operation, and the results are shown in Figure 10. As can be seen in Figure 10b, the first resonance occurs at ~6.3 KHz.

The profilometer measurements of the hot-plate shape showed that the stage is concaved with the center being lower than the corners by 5 µm. The measurement does not agree with Figure 10b. However, it makes sense since the BOX introduces compressive stresses, which are released by increasing the surface area on the bottom. The tensile stresses which develop compensate the compressive stresses. It is evident that the mechanical simulation requires a model which takes into consideration the vertical gradients of the internal stresses.

### 3.4. Measurement and Discussion

**Figure 11:**
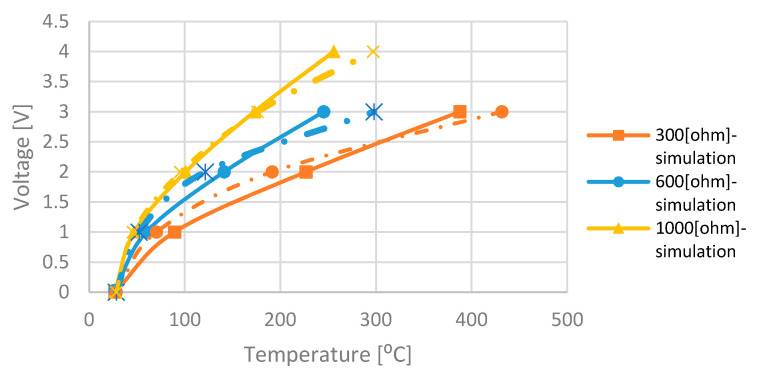
Simulation (solid lines) and measurements (dashed lines) results. Applied heater voltage vs. the average hot-plate temperature for different heater resistor. Note: Measured temperatures for high heater voltages (4–5 V) were ignored in this figure because the sub-threshold assumption is not valid at such *Vgs* values and for resistor of 300 ohms.

The drawbacks in the measurements modeling are the facts that some values such as *I*_0_ and *V_T_*_0_ are extracted from the experimental log *I_DS_*-*Vgs* curve and can cause inaccuracies as well as the inability to extract the temperature when high heater power is applied because the subthreshold formulas are no longer valid (see Figure 8). Even with those drawbacks, Figure 11 exhibits the simulations and measurements results. There is a good correlation between the results.

## 4. Reducing the Total Consumed Power during Operation by Applying Duty Cycle Operation

The total consumed power consists of the power consumed by the thermal sensor namely the TMOS transistor and the power consumed by the heater for operation and refreshment cycles. The thermal sensor of the GMOS gas sensor of Parts 1 and 2 is a micro-machined CMOS transistor operating at subthreshold (dubbed TMOS). The operation point is typically with *Vgs*~1 V and current up to 10 µA, hence they require very low power. The main required power is for the heating resistor and consists in turn of the power needed for the ignition temperature and the power needed for the periodic refreshment procedures. For ignition, a heating to about 200 °C is needed, so the heating power is about 10 mWatt (as can be estimated using simulation data of $G_{th}$:$P_{Joule-heating}=175\cdot G_{th}$ or calculating $P_{Joule-heating}$ = *V*^2^*/R*(*T*)). For the refreshment, the Joule heating power is of the order of 20 mWatt because a heating to 400 °C is required. To achieve 400 °C, we preferred to use devices with heaters having resistance of 300 ohm and 600 ohm and applied voltage of 3.3 V and 4.5 V, respectively. Since overall $P_{Joule-heating}=I\cdot V∼30\;\mathrm{mWatt}$, the average current can be estimated as 10 mA. Such high currents are unaccepted for battery operation. Hence, the GMOS should be operated by reduced duty cycle.

**Figure 12:**
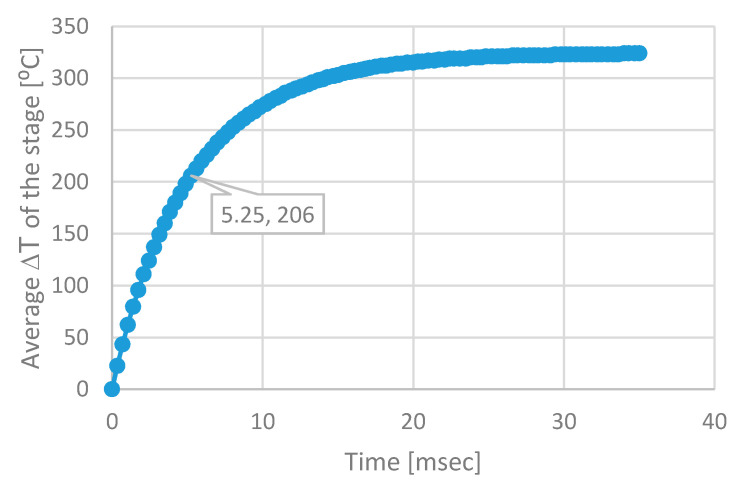
Transient simulation of the DUT (plate area 213.2 µm × 213.2 µm, arm width 6.4 µm and a gap of 18 µm), applied heater voltage of $V_{heater}=5\;V$, and $R_{heater}=1000\;\mathrm{ohm}$. The thermal time constant is 5.25 msec.

**Figure 13:**
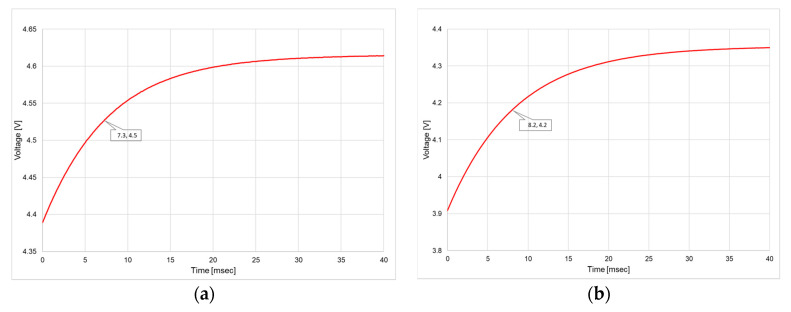
Transient measurements of the DUT voltage response to heating: (**a**) Current 4.2 mA is supplied to transistor; the thermal time constant is 7.3 msec. (**b**) Current 2.5 mA is supplied to heater; the thermal time constant is 8.2 msec. (Plate area 213.2 µm × 213.2 µm, arm width 6.4 µm and a gap of 18 µm, $R_{heater}=1000\;\mathrm{ohm}$).

To prove this opportunity, the thermal time constant *τ* of GMOS was simulated (Figure 12) and measured (Figure 13) to estimate the time needed to reach the desired temperature of the device.

The temperature of GMOS is changing exponentially with time in case of a constant Joule heat:

(12) $$\Delta T\left(t\right)=\frac{P_{Joule}}{G_{th}}\left(1-exp\left(-\frac{t}{\tau }\right)\right)$$

So, the sensor voltage response is changing accordingly:

(13) $$\Delta V\left(t\right)=\frac{dV}{dT}\Delta T\left(t\right)=\frac{dV}{dT}\cdot \frac{P_{Joule}}{G_{th}}\left(1-exp\left(-\frac{t}{\tau }\right)\right)$$

The thermal time constant *τ* is determined from the measurement as exponential fit parameter. Time dependencies were measured by means of semiconductor device analyzer (SDA) B1500A. Two types of experiments have been done:

- Current was supplied to the transistor in 2T mode (drain and gate were shorted); heater did not operate (Figure 13a).
- Current was supplied to the heater; transistor did not operate (Figure 13b).

The pulse operation is indeed feasible since the thermal time constant τ of the GMOS is of the order of few milliseconds, as shown by simulation (Figure 12) and confirmed by measurements (Figure 13). By operating at reduced duty cycle and applying the refreshment heating pulse with a duty cycle of 1%, namely every 100 sec for 1 sec, the power is reduced to 300 µWatt. The sensing duty cycle can also be optimized. In the Part 1 [16], a 50% duty cycle of 500 ms was used resulting in the average power of 5 mWatt. It can be further reduced by taking into account the small time constant exhibited in Figure 12 and Figure 13. However, the time needed for the data readout as well as for the stabilization of the gas combustion reaction should be also considered. In fact, a 10% duty cycle of 100 ms is quite reasonable leading to further reducing of the average power to 1 mWatt. By taking a reading every 10 s, the power may be further reduced. Thus, the total average power for the sensing and the refreshment can be less than 1 mWatt. Then, the corresponding average battery current is about 100 µA, which is acceptable for a battery operation. However, although the sensor, heater and readout circuitry require low power, the MCU (microcontroller) on the board of the sensing system consumes relatively high power. Therefore, battery and wireless operation require careful programming of the sensing system, applying “sleeping mode” operation to the MCU. Thus, the entire sensing board may be battery operated although the battery life time is shortened by the MCU.

## 5. Conclusions

The advantages of the innovative GMOS gas sensor are outlined in Part 1 and in Ref. [12]. These advantages of GMOS allow fabricating low-cost gas sensor that requires low power, and make it a promising technology for future smartphones, wearables, and IoT applications. The present paper emphasizes on the role of a careful design based on MEMS advanced simulations. Moreover, it exhibits the need to take into consideration the designing of the overall physical aspects, including thermal, electrical, mechanical, and power requirements.

The heart of the sensing system is the sensor. However, the users require high performance achieved by the entire system, rather than that of just the sensor. Therefore, battery and wireless operation require careful programming of the entire sensing system residing on a board. The design of the entire sensing system based on the GMOS will be reported elsewhere.

## Figures and Tables

**Table 1:** Materials thermal and mechanical properties used in this article simulations. The thin film silicon device layer is assumed to have the thermal conductivity of polysilicon rather than bulk crystalline silicon.

Property	Description	SiO_2_	Poly Si	Si(c)	Tungsten	Platinum	Silicon Nitride
*k* [W/(mK)]	Thermal conductivity	1.4	40	40	173	21,450	31
*C_P_* [J/(kgK)]	Heat capacity at constant pressure	730	700	700	134	126	1100
ρ [kg/m^3^]	Density	2200	2320	2329	19,300	71.6	3250
*E* [GPa]	Young’s modulus	70	160	-	411	-	-
ν	Poisson’s ratio	0.17	0.22	-	0.28	-	-

**Table 2:** Typical values for the CMOS-SOI process of the fabricated DUT.

Study Parameters	Value	Units
$\mu C_{ox}$	$2.8\times 10^{-5}$	$\frac{A}{V^{2}}$
*N*	2	-
*dV_t_*/*dT*	$-2.5\times 10^{-3}$	$\frac{V}{K}$
$V_{T}\left(T_{0}\right)$ (from the graph when $V_{heater}=0$)	1.3	V
$T_{0}$	300	K
*W*/*L*	1425	-
$R_{heater}$	1000	Ω
*I*_0_ (extracted from Figure 7)	$1.187\times 10^{-5}$	A

**Table 3:** The plate temperature as a function of heater voltage evaluated from the measurements of Figure 8.

*V_heater_* (V)	0	1	2	3	4	5
Temperature (K)	301.44	318.90	368.34	449.01	570.29	774.44
